# Supplementary material for: Androgen and glucocorticoid receptor direct distinct transcriptional programs by receptor-specific and shared DNA binding sites
Source: Nucleic Acids Res. 2021 Mar 22;49(7):3856–75. doi: 10.1093/nar/gkab185 (PMC8053126; doi:10.1093/nar/gkab185)
Supplement: gkab185_Supplemental_Files [file gkab185_supplemental_files.zip › Borschiweretal_16022021_SupplementaryFigs&Tables.pdf]

Supplementary Figures and Tables

Androgen and glucocorticoid receptor direct distinct transcriptional programs by receptor-specific and shared DNA binding sites

Marina Kulik[1#], Melissa Bothe[1#], Gözde Kibar[1], Alisa Fuchs[1], Stefanie Schöne[1], Stefan Prekovic[2], Isabel Mayayo Peralta[2], Ho-Ryun Chung[1,3], Wilbert Zwart[2,4], Christine Helsen[5], Frank Claessens[5], Sebastiaan H. Meijsing[1,6*]

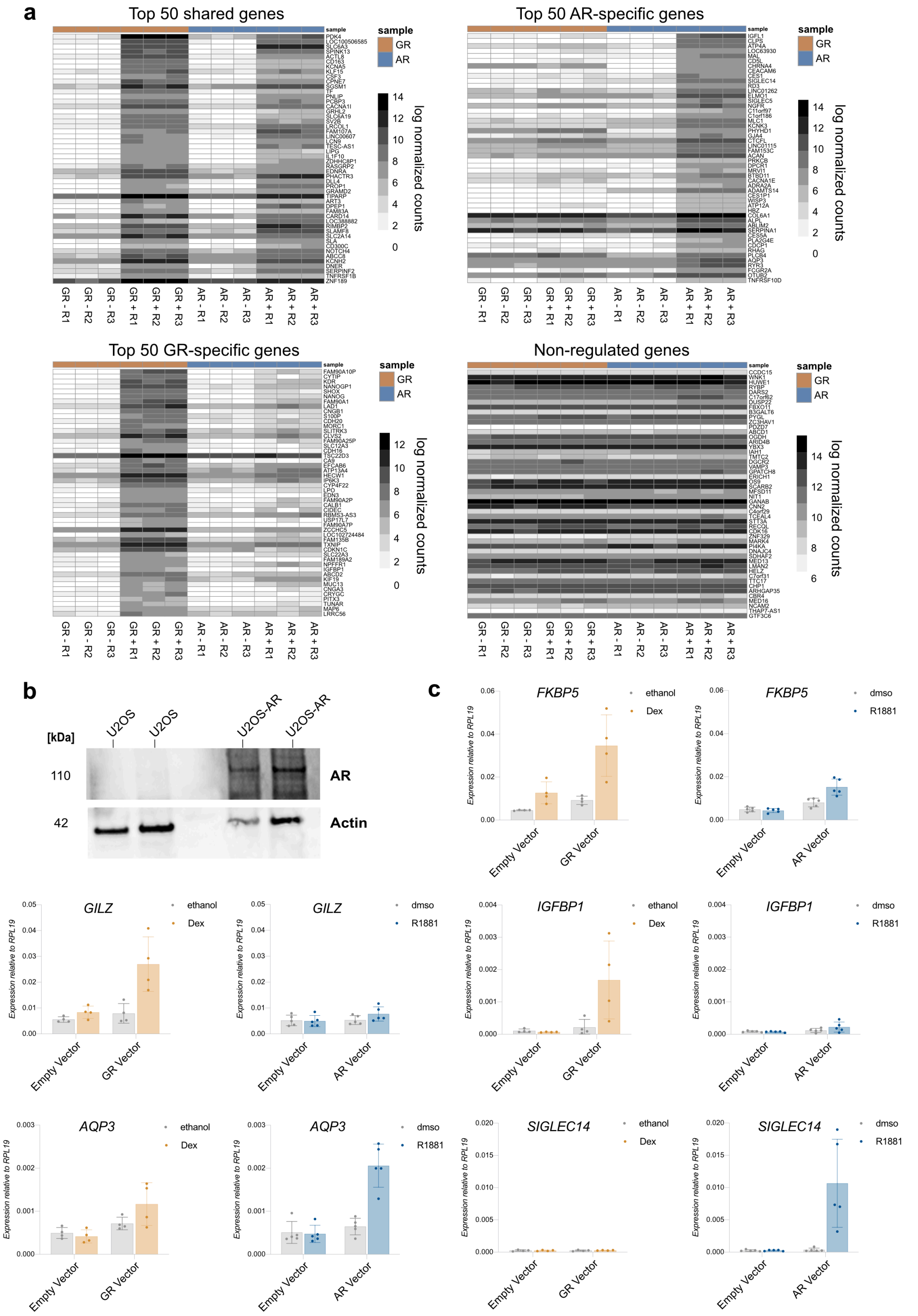

Fig. S1. Analysis of the U2OS-AR cell line and heatmaps of different gene classes (a) Heatmap displaying log normalized gene expression for the top 50 shared target genes (adjusted p-value < 0.05 and log2(fold change) > 1.5), top 50 AR-specific genes, top 50 GR-specific genes or 50 randomly selected non-regulated genes (adjusted p-value < 0.5 and 0.5 > log2(fold change) > 0). Each column represents an individual replicate sample of either AR or GR (GR -, GR +, AR -, AR+), with rows representing individual genes. U2OS-AR cells were treated for 24h with 5 nM R1881; U2OS-GR cells were treated for 4h with 1 µM Dex. (b) Total cell extracts of parental U2OS cells and a single-cell-derived U2OS-AR line were probed by Western blotting for the expression of AR and actin as loading control. (c) Relative mRNA levels of the shared target gene *FKBP5* (d) the GR-specific target genes *GILZ* and *IGFBP1* or (e) the AR-specific target genes *AQP3* and *SIGLEC14* was quantified by qPCR for U2OS cells transiently transfected with plasmid as indicated. To assay regulation by GR, cells were treated with ethanol as vehicle control or 100nM Dex for 24h. For AR for 24h with dmso as vehicle control or 5 nM R1881. Average gene expression ±SD is shown (n ≥ 3).

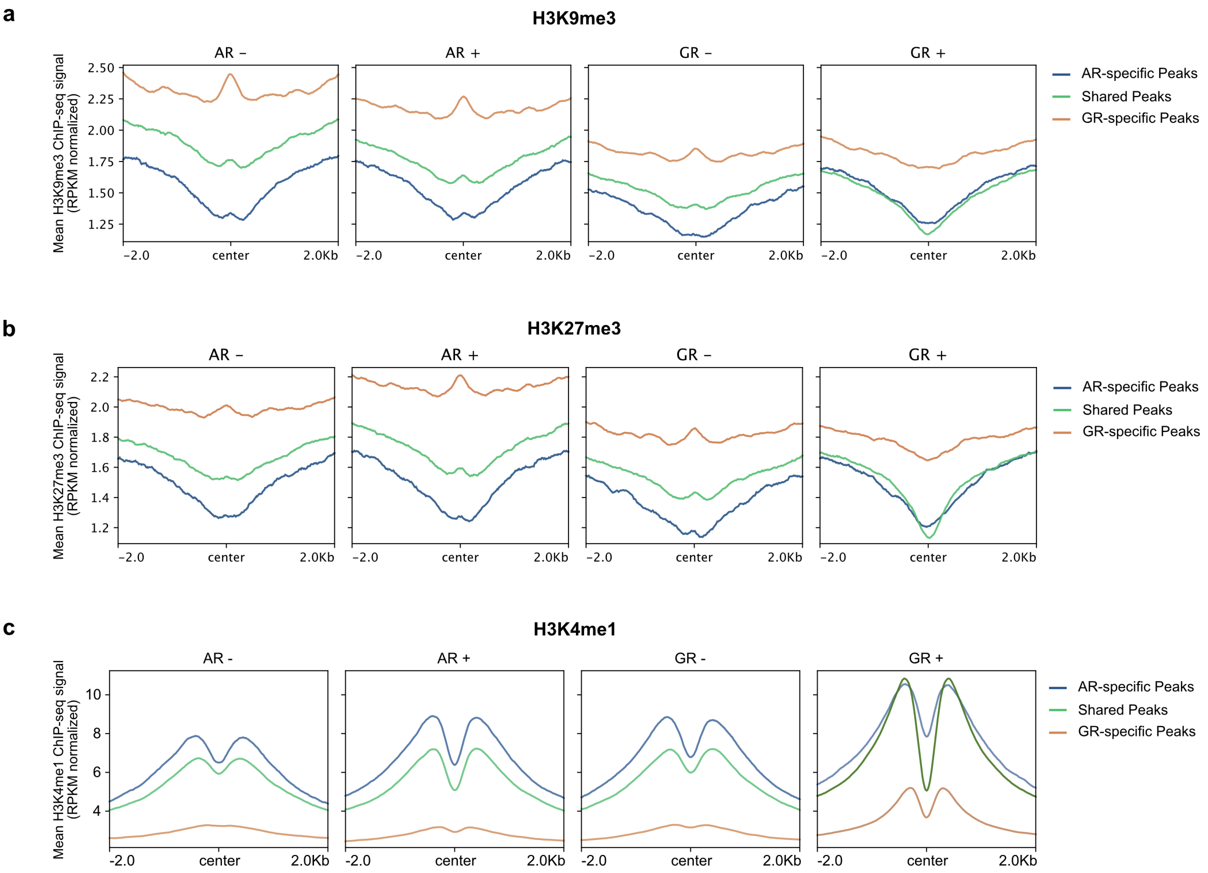

Fig. S2. Mean signal plots for chromatin marks at shared and receptor-specific peaks. Mean signal plot of (a) H3K9me3, (b) H3K27me3 and (c) H3K4me1 ChIP-seq read coverage (RPKM normalized) at shared and receptor-specific binding sites (+/- 2 kb around peak center). U2OS-AR cells were treated with R1881 or vehicle (5 nM, 4h) and U2OS-GR cells with Dex or vehicle (1 µM, 1.5h).

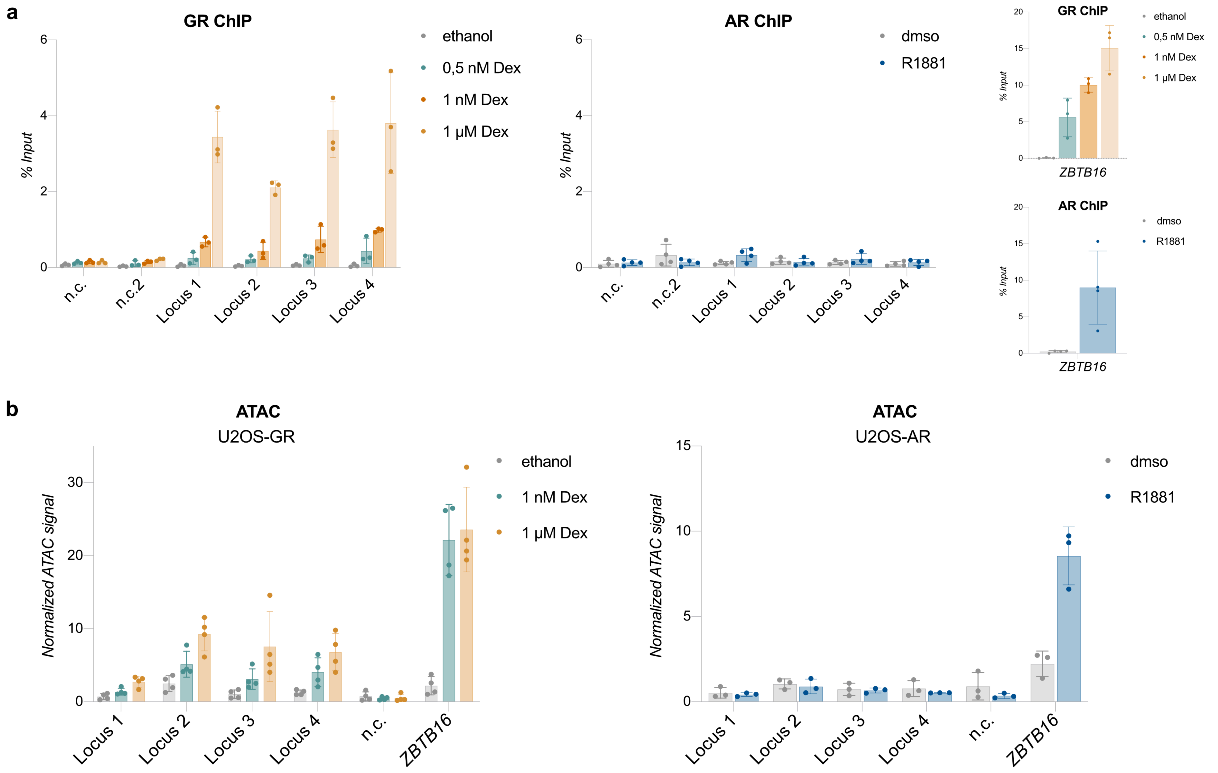

Fig. S3. GR binding and ATAC signal at low hormone concentrations. (a) ChIP-qPCR of GR-specific peaks as indicated in U2OS-GR and U2OS-AR cells. U2OS-GR cells were treated with 0.5 nM, 1 nM, 1 μM Dex or ethanol as a vehicle control for 1.5h. U2OS-AR cells were treated with 5 nM R1881 or dmso for 4h. Average percentage of input precipitated ± SD is shown (n ≥ 3). n.c.: negative control region Right: binding of either GR or AR at a shared binding site near the *ZBTB16* gene. (b) Same as for (a) except that ATAC-qPCR signal normalized to genomic DNA is shown.

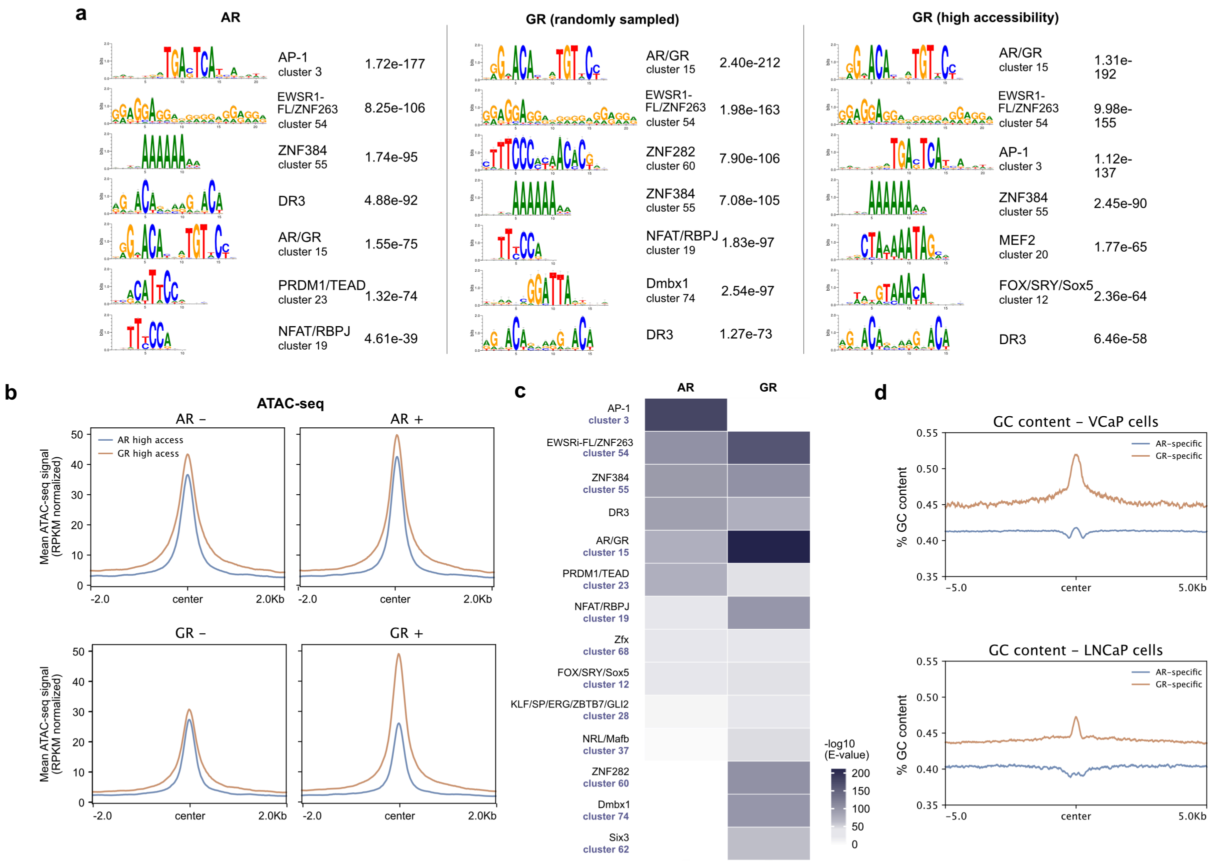

Fig. S4. Sequence motif analysis. (a) Top 7 enriched motif clusters and corresponding E values at all AR-specific peaks, an equal number of randomly sampled GR-specific peaks or GR-specific peaks in regions with high chromatin accessibility (+/- 250 pb around the peak center). Shuffled input sequences were used as background for the motif enrichment analysis. (b) Mean signal plots of ATAC-seq read coverage (RPKM normalized) at high accessibility AR- and GR-specific sites as used in Fig. 4C (+/- 2 kb around peak center). U2OS-AR cells were treated with vehicle or R1881 (5 nM, 4h) and U2OS-GR cells with vehicle or Dex (1 µM, 1.5h).(c) Heatmap visualization of enriched motif clusters (from JASPAR 2018 CORE Vertebrates Clustering motifs) at all AR-specific or at an equal number of randomly sampled GR-specific peaks (+/- 250 pb around the peak center). Shuffled input sequences were used as background for the motif enrichment analysis. Motifs were included if the E value was $<10^{-30}$ for either AR or GR. (d) GC content at GR- and AR-specifically occupied regions in VCaP and LNCaP cells (+/- 5 kb around the peak center).

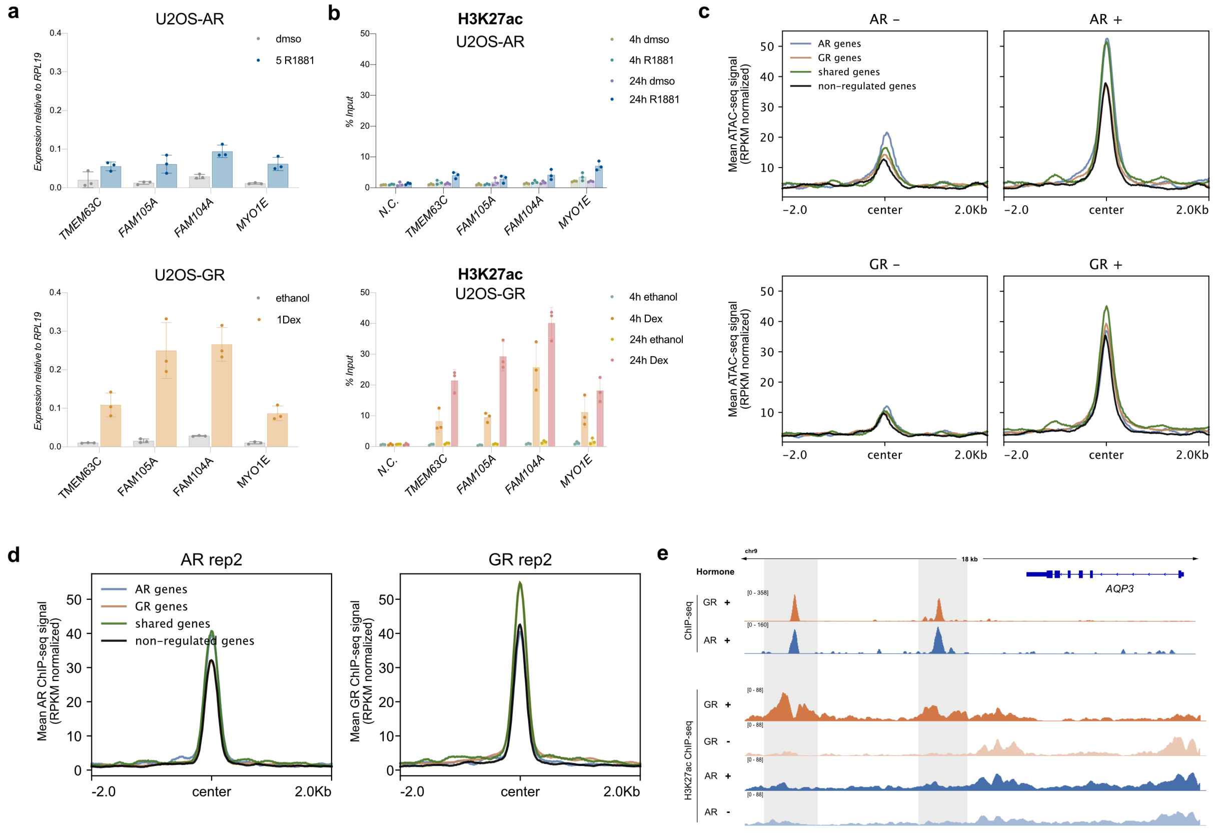

Fig. S5. Shared regulation, receptor and H3K27ac ChIP-seq and ATAC-seq at shared binding sites. (a) Relative mRNA levels of *TMEM63C*, *FAM105A*, *FAM104A* and *MYO1E* were quantified by qPCR for U2OS cells stably expressing either (top) AR or bottom (GR). U2OS-AR cells were treated for 24h with dmso as vehicle control or 5 nM R1881. U2OS-GR cells with ethanol as vehicle control or 1 µM Dex for 24h. Average gene expression ±SD is shown (n = 3). (b) H3K27ac levels were analyzed by ChIP for regions occupied by both AR and GR near the shared target genes as indicated. U2OS-AR cells were treated for either 4h or 24h with dmso as vehicle control or 5 nM R1881. U2OS-GR cells were treated for either 4h or 24h with ethanol as vehicle control or 1 µM Dex. Average percentage of input precipitated ± SD is shown (n = 3). n.c.: negative control region. (c) Mean signal plot of ATAC-seq read coverage (RPKM normalized) at shared sites (+/- 2 kb around the peak center) near the different gene categories as used in Fig. 5C. U2OS-AR cells were treated with R1881 or vehicle (5 nM, 4h) and U2OS-GR cells with Dex or vehicle (1 µM, 1.5h). (d) Same as (c) except that AR and GR ChIP-seq read coverage (RPKM normalized) is shown. (e) Genome browser screenshot for the *AQP3* locus showing receptor and H3K27ac ChIP-seq signals for cells treated as indicated. Regions bound by both receptors are highlighted in grey.

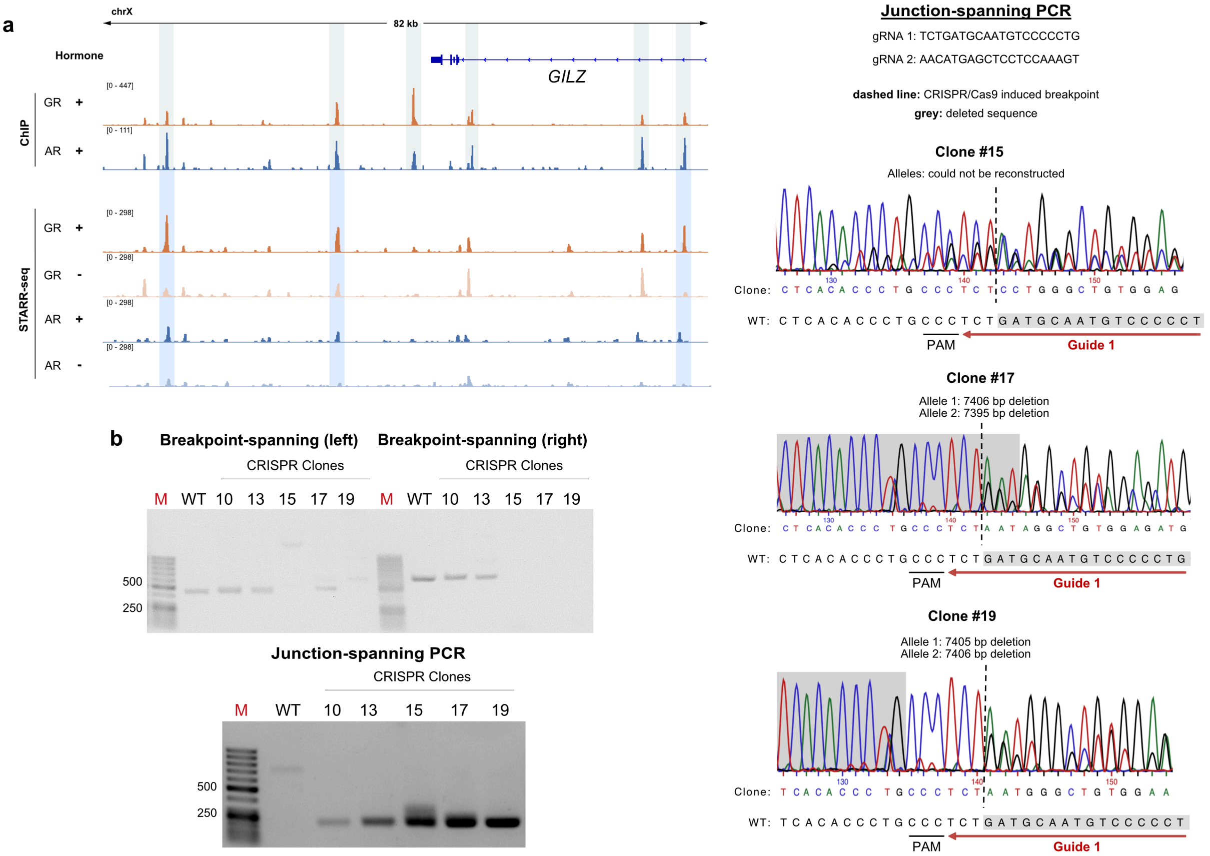

Fig. S6. Receptor binding and FAIRE-STARR signal at the *GILZ* locus/genotyping of clonal lines. (a) Genome browser screenshot of the *GILZ* locus showing ChIP-seq and STARR-FAIRE-seq tracks for GR and AR. The STARR-seq tracks show enhancer activity in U2OS-GR cells treated with ethanol or 1 µM Dex and U2OS-AR cells treated with dmso or 5 nM R1881 overnight. The receptor-bound peaks are highlighted in grey and hormone-inducible STARR-seq enhancers are highlighted in blue. STARR-seq tracks depict the merged signal from three biological replicates. (b) Left: To genotype single-cell-derived U2OS-AR clonal lines, the CRISPR-targeted *AQP3* locus was PCR amplified from genomic DNA and analyzed on agarose gels. For the junction-PCR, primers are placed outside the breakpoints to detect CRISPR clones carrying a genomic deletion at the *AQP3* locus. Primers flanking the breakpoints were used to detect the presence of WT alleles. The expected amplicon size of the left WT breakpoint is 476 bp and 686 bp for the right WT breakpoint. For clone #17, a signal is observed for the left WT breakpoint of clone #17 indicating a partial deletion of the second allele. M represents the DNA size marker GeneRuler 50bp. Right: Sanger sequencing results of the junction-PCR amplicon for successfully edited clonal lines #15, #17 and #19 and the inferred deletion for each allele.

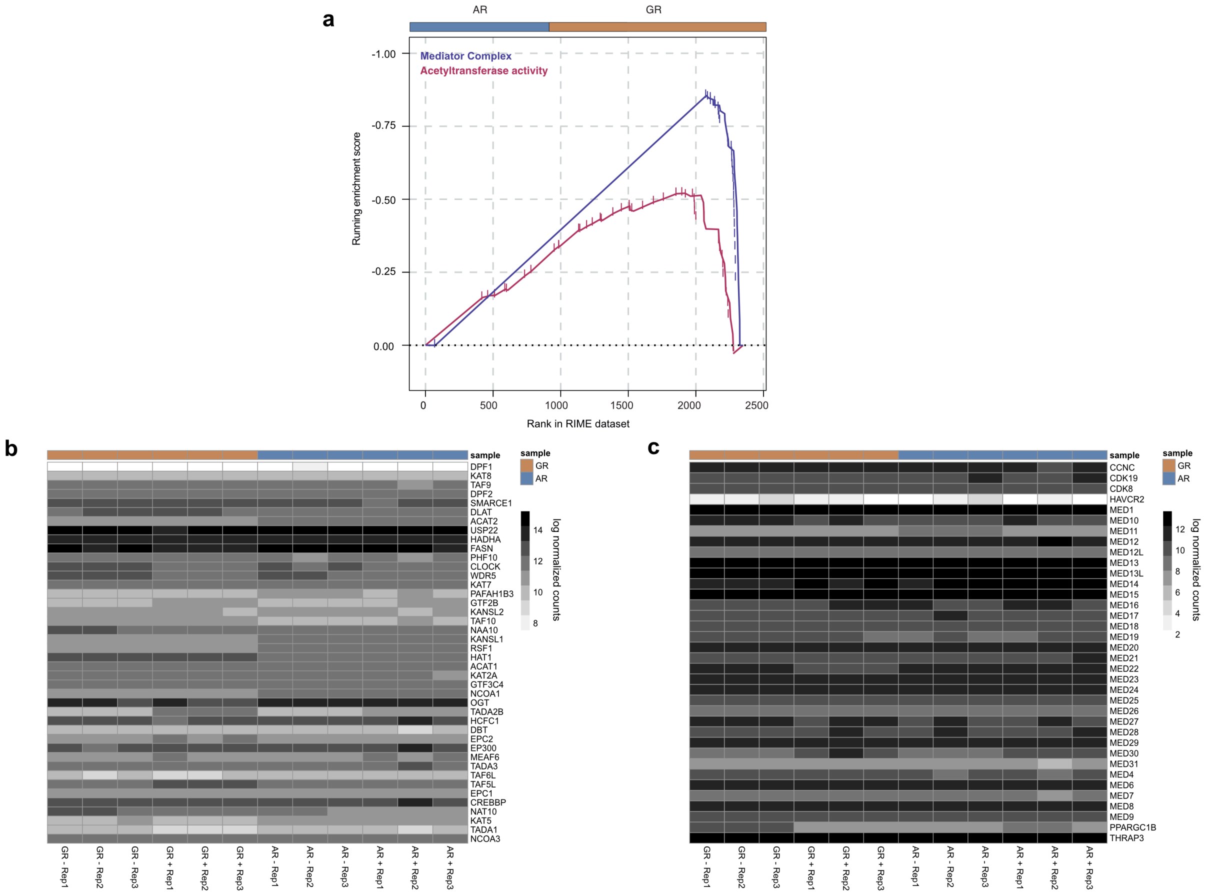

Fig. S7. Gene Set Enrichment Analysis of the RIME data. (a) Gene Set Enrichment Analysis (GSEA) results comparing the RIME data for AR vs GR. Two pathways with GR-specific enrichment are shown. (b) Heatmap displaying log normalized gene expression for proteins from the acetyltransferase activity category that selectively interacted with GR. U2OS-AR cells were treated for 24h with 5 nM R1881; U2OS-GR cells for 4h with 1 µM Dex. (c) Same as for (b) except for genes of the mediator complex category.

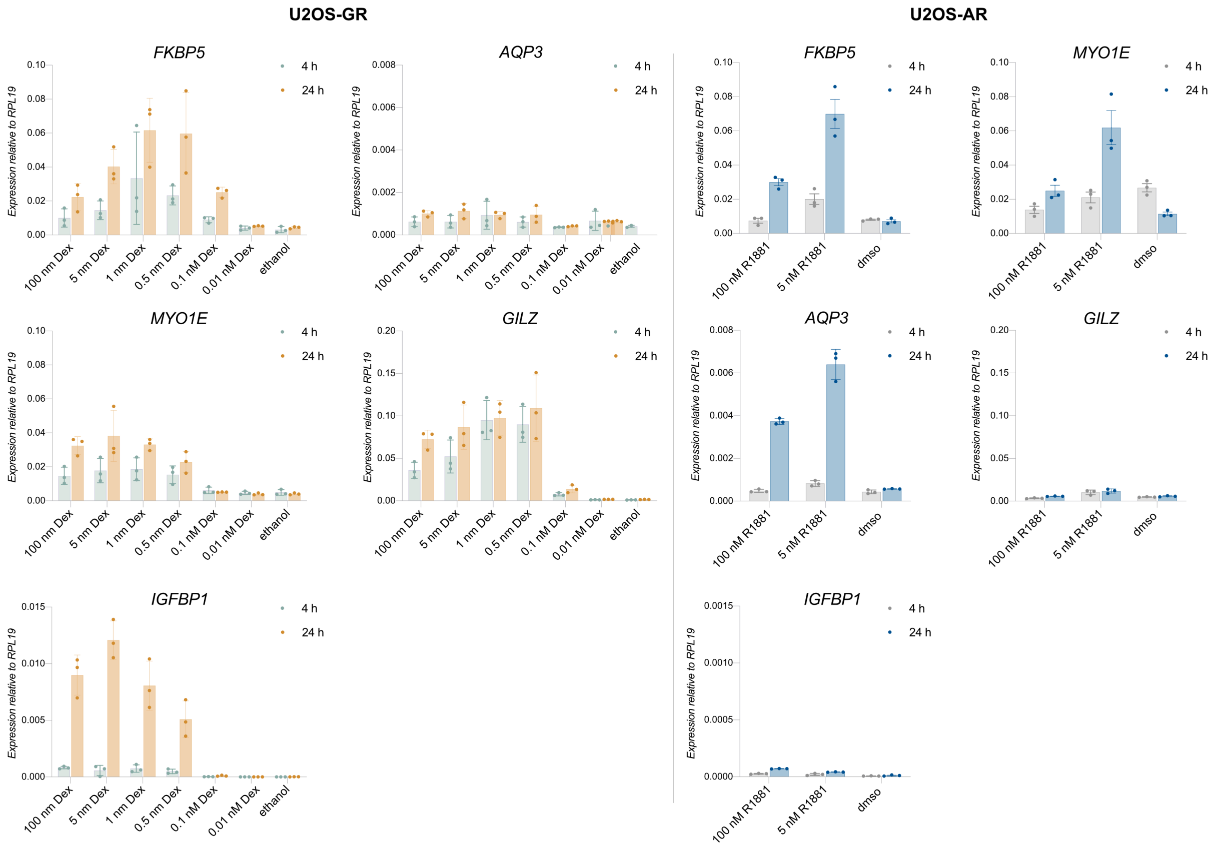

Figure S8. Gene regulation at different hormone concentrations and time points. (a) Relative mRNA levels of genes as indicated was quantified by qPCR for U2OS cells stably expressing either (left) GR or (right) AR. U2OS-AR cells were treated for 4h or 24h with dmso as vehicle control or with R1881 concentration as indicated. U2OS-GR cells were treated with ethanol as vehicle control or with Dex concentration as indicated for 4h or 24h. Average gene expression ±SD is shown (n = 3).

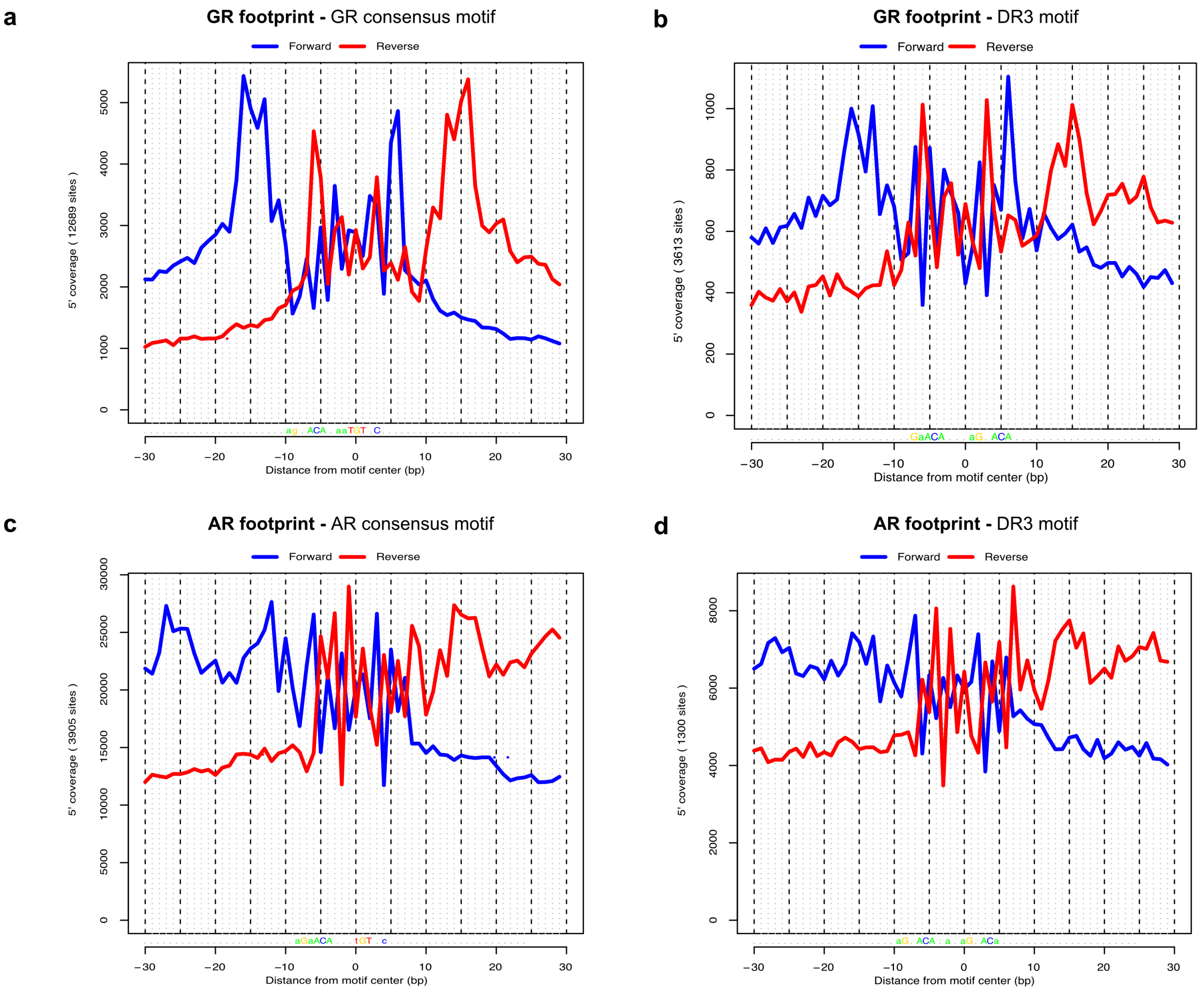

Figure S9. ChIP-exo profiles for AR and GR at canonical and at direct repeat-like (DR3) sequences. (a) GR ChIP-exo footprint profile for the GR consensus motif in U2OS-GR cells. Blue represents ChIP-exo signal for the positive and red the negative strand. (b) GR ChIP-exo footprint profile for the DR3 motif in U2OS-GR cells. (c) AR ChIP-exo footprint profile for the AR consensus motif in LNCaP cells. (d) AR ChIP-exo footprint profile for the DR3 motif in LNCaP cells.

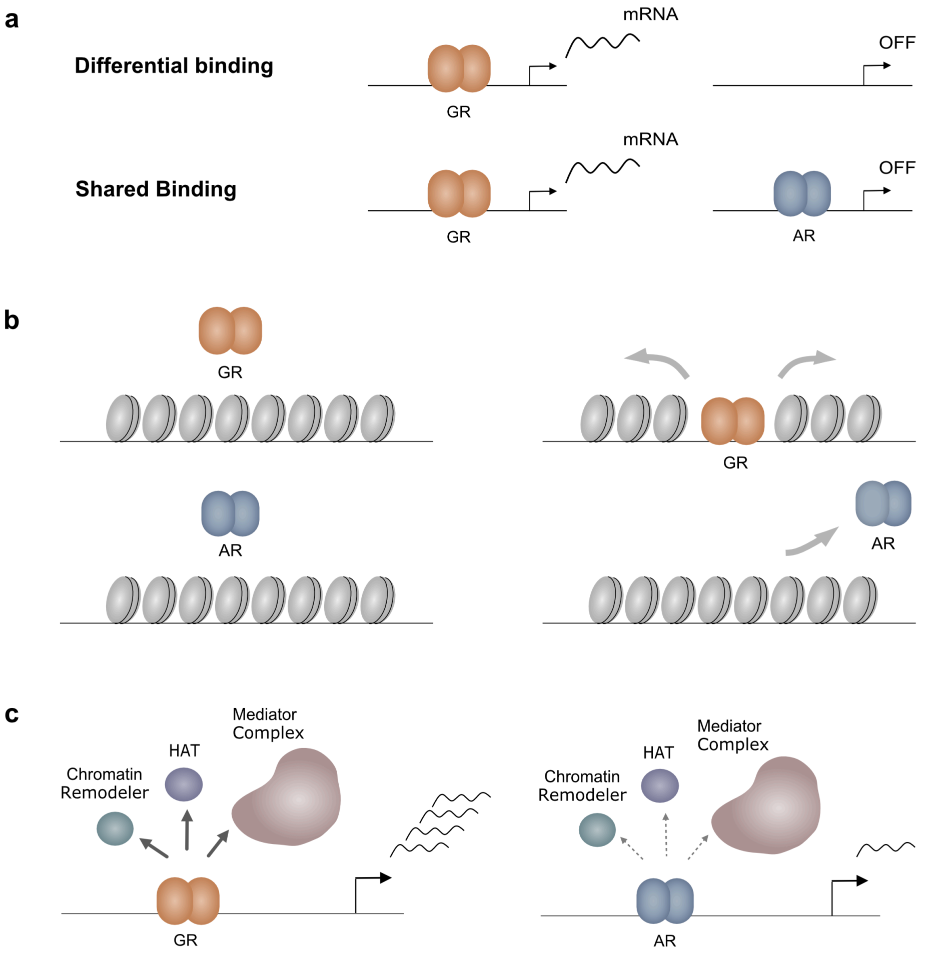

Fig. S10. Models explaining receptor-specific regulation. (a) Specificity of transcriptional regulation can be driven by both receptor-specific binding and by selective activation from binding sites that are occupied by both AR and GR. (b) Receptor-specific binding due to a GR-specific ability to bind relatively inaccessible chromatin. (c) Receptor-specific interactions with cofactors can drive receptor-specific regulation from shared binding sites.

Table S1: Regulatory regions of individual STARR-seq constructs

Invariant sequence for In-Fusion cloning highlighted in bold. Mutated positions underlined.

Positive control *IP6K3*
TAGAGCATGCACCGGACACTCTTTCCCTACACGACGCTCTTCCGATCTTGGGATAATCTGCCCAAGGTCACATGGTCGGTGTGGGGT
GGAGCTCTGGGAGGGCCATGCCAGACGTGGGGATGGAGGAAAGGACAGGCTGTTCCAGAAGGTCTGGTGTTGTCACGCTGTTTACCC
CAAGTTTGCATTAGGGACATTCCTGCTGTGTCCCCTGTCATGTTTGTTCTGGTCATTCTGTTCCATAGGAGAGAACAGAGACGCTGTGAT
TCCCTCCCCTCAGGGAGGGTCGTCAGCGCTGAGGGCTGGGAGCCCAGGCTGCAAGGAGATGGTGTTTACATTTCCAGGCCTGTGTCTT
GGGGAAGGAGGATTGGAGTGCTTTGTTCTAGGGAGGAGAACTGACCAGATCGGAAGAG**CACACGTCTGAACTCCAGTCACTCGACG**
AATTCGGCC

AQP3-enhancer wt
TAGAGCATGCACCGGACACTCTTTCCCTACACGACGCTCTTCCGATCTTGGAGTAGTCCCTCTTCTCCCCTTCCCACCCCACCC
AACTCTACCCCACCCACGTTTCCCCAAGCCTAGAAGTGCCCCACCTAGGTTTTCAGAAAAACAAAATCAAGAGGAAAGAGGAAG
GAGGGAGCTCATTACCAGGAATTAAGGAGGAGGCCTGCCCTAGGGGAACACAAGTGAGACTTGGCTGGCAGGCAGAGATAT
GCACTGAGTACATGGAGGTGTACTCAAAGTACACTGAGAGAGGGGCTCAGAGGAACATGCAGAGATGTGATCTGAGGGTCAC
AGTCCAGGATGTTCACTCAGAGGTACACAAAGAAAGACCCAGACACAAGGCTGGGCACTGTGGTTCATGCCTGTAATCCCAGC
ACTTTGGGTGGCCAAGGCAGGTGGATCACCTGAGGTCAGGAGTTCGACACCAGCCTGGGCAACACGGTGAAACCCTGTCTCT
ACTAAAAATACAAAAATTAGCTGGGCGTGGTGGCATGTGCTTGTAATCCCAGCTACTCGGGAGGCTGAGGCAGGAGAATTGC
TTCAACCCAGGAGGCAGAGGTTGC**CACACGTCTGAACTCCAGTCACTCGACGAATTCGGCC**

AQP3-enhancer mutated
TAGAGCATGCACCGGACACTCTTTCCCTACACGACGCTCTTCCGATCTTGGAGTAGTCCCTCTTCTCCCCTTCCCACCCCACCC
AACTCTACCCCACCCACGTTTCCCCAAGCCTAGAAGTGCCCCACCTAGGTTTTCAGAAAAACAAAATCAAGAGGAAAGAGGAAG
GAGGGAGCTCATTACCAGGAATTAAGGAGGAGGCCTGCCCTAGGGGAACACAAGTGAGACTTGGCTGGCAGGCAGAGATAT
GCACTGAGTACATGGAGGTGTACTCAAAGT<u>ATA</u>CTG<u>ATA</u>GAGGGGCTCAGAGGA<u>ATA</u>TGC<u>ATA</u>GATGTGATCTGAGGGTCAC
AGTCCAGGATGTTCACTCAGAGGT<u>ATA</u>CAA<u>ATA</u>AAGACCCAGACACAAGGCTGGGCACTGTGGTTCATGCCTGTAATCCCAGC
ACTTTGGGTGGCCAAGGCAGGTGGATCACCTGAGGTCAGGAGTTCGACACCAGCCTGGGCAACACGGTGAAACCCTGTCTCT
ACTAAAAATACAAAAATTAGCTGGGGCCACCACGATGTGCTTGTAATCCCAGCTACTCGGGAGGCTGAGGCAGGAGAATTGCT
TCAACCCAGGAGGCAGAGGTTGC**CACACGTCTGAACTCCAGTCACTCGACGAATTCGGCC**

AQP3-enhancer AGA->TGT
TAGAGCATGCACCGGACACTCTTTCCCTACACGACGCTCTTCCGATCTTGGAGTAGTCCCTCTTCTCCCCTTCCCACCCCACCC
AACTCTACCCCACCCACGTTTCCCCAAGCCTAGAAGTGCCCCACCTAGGTTTTCAGAAAAACAAAATCAAGAGGAAAGAGGAAG
GAGGGAGCTCATTACCAGGAATTAAGGAGGAGGCCTGCCCTAGGGGAACACAAGTGAGACTTGGCTGGCAGGCAGAGATAT
GCACTGAGTACATGGAGGTGTACTCAAAGTACACTG<u>TGT</u>GAGGGGCTCAGAGGAACATGC<u>TGT</u>GATGTGATCTGAGGGTCAC
AGTCCAGGATGTTCACTCAGAGGTACACAA<u>TGT</u>AAGACCCAGACACAAGGCTGGGCACTGTGGTTCATGCCTGTAATCCCAGC
ACTTTGGGTGGCCAAGGCAGGTGGATCACCTGAGGTCAGGAGTTCGACACCAGCCTGGGCAACACGGTGAAACCCTGTCTCT
ACTAAAAATACAAAAATTAGCTGGGGCCACCACGATGTGCTTGTAATCCCAGCTACTCGGGAGGCTGAGGCAGGAGAATTGCT
TCAACCCAGGAGGCAGAGGTTGC**CACACGTCTGAACTCCAGTCACTCGACGAATTCGGCC**

Table S2: Primers to clone sgRNAs targeting the AQP3 locus

Primer name	Sequence:	Genomic coordinate target (hg19)
AQP3_214_up_fwd	CACCGCAGGGGGACATTGCATCAGA	Chr9:33430752-33430772
AQP3_214_up_rev	AAACTCTGATGCAATGTCCCCCTGC	Chr9:33430752-33430772
AQP3_266_down_fwd	CACCGAACATGAGCTCCTCCAAAGT	Chr9:33438144-33438163
AQP3_266_down_rev	AAACACTTTGGAGGAGCTCATGTTC	Chr9:33438144-33438163

Table S3: Primer pairs for qPCR:

<u>cDNA:</u>	
hRPL19-fw:	ATGTATCACAGCCTGTACCTG,
hRPL19rev:	TTCTTGGTCTCTTCCTCCTTG,
GFP-fw:	GGCCAGCTGTTGGGGTGTC,
GFP-rev:	TTGGGACAACTCCAGTGAAGA
GILZ-fw:	*CCATGGACATCTTCAACAGC*
GILZ-rev:	*TTGGCTCAATCTCTCCCATC*
AQP3-fw:	*GCAGCCTGTCCATCTGTG*
AQP3-rev:	*ACCCTACTTCCCAAAAGCC*
FKBP5-fw:	*TGAAGGGTTAGCGGAGCAC*
FKBP5-rev:	*CTTGGCACCTTCATCAGTAGTC*
ABLIM3-fw:	*TATTAGTCCACGCGCCTTCA*
ABLIM3-rev:	*TGCTGATAAGGAATGCTAGTGT*
IGFBP1-fw:	*TCACAGCAGACAGTGTGAGAC*
IGFBP1-fw:	*AGACCCAGGGATCCTCTTC*
SIGLEC14-fw:	*TGGAGGTGACAGCCCTGATA*
SIGLEC14-rev:	*GAATGTGAGAGGTGGTCCCG*
TMEM63C-fw:	*GTCTGGGGTCACTCTTCTGC*

TMEM63C-rev:	*AGAGCAACCCAAAAGGCACA*
FAM105A-fw:	*TGTGGAGGCAGAGGTTGATTT*
FAM105A -rev:	*AGCTCCTCATAAGCCTTCCTC*
FAM104A-fw:	*GTAGTGTCAGCGGGCATCTT*
FAM104A -rev:	*GGCGGCGCAATAGAGAAGTA*
MYO1E-fw:	*AAGACCGTCCGGAACAACAA*
MYO1E -rev:	*GCCCTCGATGAGCTGGTAAA*

<u>*ChIP* / ATAC:</u>

n.c1-fw:	AATGGCAGCCCCTAGTCATTC
n.c1-rev:	AACTGGGAGTGATACTGGTTCC
n.c2-fw:	TGCATGACGCAGACCTTTCT
n.c2-rev:	ATGAGAACCACATGGGCCAG
locus1-fw:	CCTTTTTCAATTTGGGTGGTT
locus1-rev:	GATGTCCATTTTCACCACGA
locus2-fw:	ATGCCACTCCCTTCTCCATT
locus2-rev:	CACAGGTCTCGGCTAACAGA
locus3-fw:	CCCATTGGTGCCAGTACTGA
locus3-rev:	AGAGGTCCGAGGTTTGAGAG
locus4-fw:	CCTCTTAGGTTGGTGCAGATT
locus4-rev:	TACCTACCCAGTTCCAGAGC
locus5-fw:	TGGGATCTGCTGACAAGTGT
locus5-rev:	CCTGTCTGCCTCCTCAAGAA
locus6-fw:	GACCGCACTTCTCAGTGTCA
locus6-rev:	GGACATCACAAACACCAGCA
locus7-fw:	ATGCAAAAGCCCCTACACAG
locus7-rev:	AGACAGCCAGAGCGTAGAGC
locus8-fw:	AGAGGTGAACGAGGTGGATG
locus8-rev:	AGGGACTTGGGAGGTCTGTT
locus9-fw:	CTTCTCTGCCCAGGTGCTAT
locus9-rev:	CTGCCACTGAAGGAGACACA
ZBTB16-fw:	CTCCTTGAGGGAAAGAACACAC
ZBTB16-rev:	ACAGACGCAGGGCATTTTAC
FKBP5-fw:	GCATGGTTTAGGGGTTCTTG
FKBP5-rev:	TAACCACATCAAGCGAGCTG

<u>ChIP Med1 / EP300: primers:</u>

GILZ Peak 1-fw:	ACTGCCTCTTTTTCTAAGGGC
GILZ Peak 1-rev:	TCTCTCATCTCATCCTCATGGA
GILZ Peak 2-fw:	CTCAGCAGCTTTTCTTCGTG
GILZ Peak 2-rev:	AACCAAGGAATTGGGTCACA
IGFBP1-fw:	CCAGGAGGTGTTTGGAATGT
IGFBP1-rev:	TCATGTTCTTAGGGGGCAAC
ABLIM3-fw:	GAGGTTTGATTCCCATTCCA
ABLIM3-rev:	CCTGGAGTGGAACACTGTGA
SRPK2-fw:	GACATCACACCTCGTCTC
SRPK2-rev:	GGATGTGCTCTTCATGTC
USP18-fw:	TGCTGGCAGAACAAGATGTC
USP18-rev:	AAGGAACCAATGTTGCTTGG

<u>ChIP H3K27ac:</u>

TMEM63C-fw:	CAAAGGGAAACCGAAGCATA
TMEM63C -rev:	CAGAGTGAAAGGCTGGGAAA
FAM105A-fw:	AAGGGGAGGAGGTGAGAGAA
FAM105A -rev:	AAGCTGGGACAGTTGGTCAC
FAM104A-fw:	GGAGTGGCTCAACAACTGAAT
FAM104A -rev:	AAAATCCGTGCATTGGTCTC
MYO1E-fw:	ACCTACAGCCATGGGTTTCA
MYO1E -rev:	TGGGCTTATCATCATCTGCA

Table S4: Primers to genotype AQP3 clonal lines

MB183_Junction_F	ACACATTTTCCCACCCCTCT
MB184_Junction_R	GGGAGAATTGCATCCCCTAT
MB185_WT_left_R	AAGCTCATCCATCACCAACC
MB187_WT_right_F2	TCCAGGATGTTCACTCAGAGG
